# Hematologists’ barriers and enablers to screening and recruiting patients to a chimeric antigen receptor (CAR) T cell therapy trial: a theory-informed interview study

**DOI:** 10.1186/s13063-021-05121-y

**Published:** 2021-03-25

**Authors:** Gisell Castillo, Manoj Lalu, Sarah Asad, Madison Foster, Natasha Kekre, Dean Fergusson, Terry Hawrysh, Harold Atkins, Kednapa Thavorn, Joshua Montroy, Stuart Schwartz, Robert Holt, Raewyn Broady, Justin Presseau, on behalf of the GO CART team

**Affiliations:** 1 Clinical Epidemiology Program, Ottawa Hospital Research Institute, 501 Smyth Road, Ottawa, Ontario K1H 8L6 Canada; 2 Blueprint Translational Research Group, Ottawa Hospital Research Institute, 501 Smyth Road, Ottawa, Ontario K1H 8L6 Canada; 3 Department of Anaesthesiology and Pain Medicine, University of Ottawa at the Ottawa Hospital General Campus, 501 Smyth Road, Ottawa, Ontario K1H 8L6 Canada; 4 Blood and Marrow Transplant Program, The Ottawa Hospital, 501 Smyth Road, Ottawa, Ontario K1H 8L6 Canada; 5 School of Epidemiology and Public Health, University of Ottawa, 600 Peter Morand Crescent, Ottawa, Ontario K1G 5Z3 Canada; 6 Patient Partners, Ottawa, Canada; 7 Institute for Clinical and Evaluative Sciences (ICES), University of Ottawa, 1053 Carling Ave., Ottawa, Ontario K1Y 4E9 Canada; 8 BC Cancer Genome Sciences Centre, 100-570 West 7th Avenue, Vancouver, British Columbia V5Z 4S6 Canada; 9 Leukemia/BMT Program, Vancouver General Hospital, 2775 Laurel St - 10th floor, Vancouver, British Columbia V5Z 1M9 Canada

**Keywords:** Chimeric antigen receptor T cell therapy, Physician screening, Barriers to trial recruitment, Theoretical domains framework, Early phase clinical trials, Early phase immunotherapy trials

## Abstract

**Background:**

Novel therapies often fail to reach the bedside due to low trial recruitment rates. Prior to conducting one of the first chimeric antigen receptor (CAR) T cell therapy trials in Canada, we used the Theoretical Domains Framework, a novel tool for identifying barriers and enablers to behavior change, to identify physician-related barriers and enablers to screening and recruiting patients for an early phase immunotherapy trial.

**Methods:**

We conducted interviews with hematologists across Canada and used a directed content analysis to identify relevant domains reflecting the key factors that may affect screening and recruitment.

**Results:**

In total, we interviewed 15 hematologists. Physicians expressed “cautious hope”; while expressing safety, feasibility, and screening criteria concerns, 14 out of 15 hematologists intended to screen for the trial (domains: knowledge, goals, beliefs about consequences, intentions). Physicians underscored the “challenging contexts,” identifying resources, workload, forgetting, and patient wait times to receive CAR T cells as key practical barriers to screening (domains: environmental context and resources, memory, attention and decision-making, behavioral regulation). They also highlighted “variability in roles and procedures” that may lead to missed trial candidates (domain: social and professional role). Left unaddressed, these barriers may undermine trial recruitment.

**Conclusions:**

This study is among the first to use the Theoretical Domains Framework from the physician perspective to identify recruitment challenges to early phase trials and demonstrates the value of this approach for identifying barriers to screening and recruitment that may not otherwise have been elicited. This approach can optimize trial procedures and may serve to inform future promising early phase cancer therapy trials.

**Trial registration:**

ClinicalTrials.gov Identifier: NCT03765177. Registered on December 5, 2018.

**Supplementary Information:**

The online version contains supplementary material available at 10.1186/s13063-021-05121-y.

## Introduction

Chimeric antigen receptor (CAR) T cell therapy is an innovative treatment for patients with hematologic malignancies [1–6]. CAR T cell therapy uses a retrovirus or lentivirus to modify T cells to express specific antigen receptors that target cancer cells when reintroduced into the body [7]. Early-phase clinical trials assessing the safety and efficacy of anti-CD19 CAR T cell therapy have found a complete response in 54% of all patients receiving the modified cells and 77% of patients with acute lymphocytic leukemia [8]. This is a considerable improvement for patients who have typically had few available treatment options [7]. However, there remain questions and concerns regarding the intricacies of CAR T cell manufacturing, storage, shipping, administration, clinical oversight, care coordination, and, most importantly, safety and efficacy, suggesting the need for further research to establish treatment safety and efficacy and refine production and processing procedures [7, 9, 10]. Thus, researchers at a Canadian urban hospital are conducting the first Canadian, investigator-led, early-phase CAR T cell therapy clinical trial, the CLIC-01 trial (ClinicalTrials.gov Identifier: NCT03765177), to address some of these open questions.

### Challenges to clinical trial recruitment

Critical to the success of the CLIC-01 trial will be the timely recruitment of eligible patients with relapsed or refractory acute lymphocytic leukemia and non-Hodgkin’s lymphoma. Unfortunately, despite some improvement in overall clinical trial recruitment rates, many novel therapies fail to reach the bedside due to low or delayed recruitment rates with early-phase trials seeing lower rates of accrual [11–13]. Both patient- and clinician-related barriers to clinical trial recruitment have been identified and documented in various systematic reviews that have emphasized a need to improve processes for identifying eligible patients, communicating trial information, obtaining consent, and ensuring participating sites have the necessary resources to support staff and prospective trial participants [11, 14–17]. Several systematic reviews have also assessed the effectiveness of tested strategies, such as training recruiters, improving consent forms and modes of information provision, the use of open trials, telephone reminders, and providing financial assistance for improving clinical trial recruitment [18–23]. Despite ongoing research on overcoming barriers to trial recruitment, no single strategy has been clearly shown to increase recruitment rates [17–21, 23]. Some have suggested that recruitment barriers are best identified and addressed in advance of a trial [13, 14, 24, 25]. More recently, qualitative studies have been conducted aimed at identifying and addressing trial-specific recruitment barriers as they unfold to mitigate recruitment challenges and have demonstrated encouraging results [26, 27].

### The role of the physician

The success of the CLIC-01 trial depends on hematologists’ willingness, opportunity, and capacity to identify and refer eligible patients to the trial. Though largely focused on later-phase randomized controlled trials, much work has been done to identify physician-related barriers that may undermine recruitment to trials. Identified barriers include physician attitudes toward clinical trials [28–30], treatment preferences [25, 28], available time and resources [24, 25, 28, 30–32], workload concerns [23, 32, 33], potential impact on patient-provider relationships [14, 28], difficulties conducting the informed consent process [14, 19, 24, 28, 34], and challenges in explaining difficult trial concepts [23, 25, 32].

Less is known about the specific barriers to early-phase cancer clinical trials despite the presence of unique factors (e.g., single-arm design, assessing treatment safety) that likely impact the recruitment process [13, 35, 36]. Studies on recruitment to early-phase clinical trials have typically focused on patient motivations and have identified beliefs in therapeutic benefits [37–39], opting for alternative treatments [40, 41], and disease progression [35] as important barriers. Other studies have noted that strict eligibility criteria [13, 42] and safety concerns [31] are barriers to recruiting for early-phase clinical trials. Despite efforts to develop training for staff involved in recruiting for early-phase trials [43], few studies have identified physician-targeted evidence-based strategies for improving recruitment to early-phase clinical trials.

### The Theoretical Domains Framework

Given the novelty, complexity, costs, and urgency for a timely evaluation of CAR T cell therapy, we sought to engage hematologists regarding anticipated recruitment barriers prior to the trial launch. For many clinicians, participating in an early-phase CAR T clinical trial represents a change from routine practice. To identify potential barriers to early-phase trial recruitment, we used the Theoretical Domains Framework (TDF) as it provides a well-tested, flexible, and comprehensive approach to identifying modifiable factors known to affect healthcare provider behavior [44–46]. The TDF is unique in that it draws on decades of behavior change research and synthesizes the constructs from 33 behavior change theories into 12 broad domains [47] that were later independently validated and expanded into 14 domains [48]. The TDF is distinct in that it allows researchers to consider theoretical explanations for identified barriers and enablers and to develop theoretically driven strategies to address them [47, 49].

We used the TDF to prospectively identify potential barriers and enablers to hematologists screening patients for an early-phase CAR T cell therapy trial. Based on the identified barriers and enablers, we aimed to make recommendations on how to optimize support, resources, and training to promote recruitment to the planned early-phase trial.

## Methods

### Design

We conducted a qualitative, theory-informed interview study to identify barriers and enablers to physicians screening for an early phase CAR T cell therapy trial.

### Interview guide development

In collaboration with the CLIC-01 trial team, we determined that hematologists were an important stakeholder group to engage prior to trial launch given their anticipated role in identifying, screening, and referring potential candidates for an early phase CAR T cell therapy trial. We used the validated version of the TDF and kept the domain that was removed from the validated version (nature of the behavior) because it captures unique information helpful in understanding participant experiences with screening behaviors. The 15 domains used in this study are presented in Table 1 along with definitions.

**Table 1:** Description of domains reproduced from the validated Theoretical Domains Framework [48]

Domain	Description
Knowledge	An awareness of the existence of something (including knowledge of condition/scientific rationale)
Skills	An ability or proficiency acquired through practice
Social/professional role and identity	A coherent set of behaviors and displayed personal qualities of an individual in a social or work setting
Beliefs about capabilities	Acceptance of the truth, reality or validity about an ability, talent, or facility that a person can put to constructive use
Optimism	The confidence that things will happen for the best or that desired goals will be attained
Beliefs about consequences	Acceptances of the truth, reality, or validity about outcomes of a behavior in a given situation
Reinforcement	Increasing the probability of a response by arranging a depending relationship, or contingency, between the response and a given stimulus
Intentions	A conscious decision to perform a behavior or a resolve to act in a certain way
Goals	Mental representations of outcomes or end states that an individual wants to achieve
Memory, attention, and decision processes	The ability to retain information, focus selectively on aspects of the environment, and choose between two or more alternatives
Environmental context and resources	Any circumstance of a person’s situation or environment that discourages or encourages the development of skills and abilities, independence, social competence, and adaptive behavior
Social influences	Those interpersonal processes that can cause individuals to change their thoughts, feelings or behaviors
Emotion	A complex reaction pattern, involving experiential, behavioral, and physiological elements, by which the individual attempts to deal with a personally significant matter or event
Behavioral regulation	Anything aimed at managing or changing objectively observed or measured actions
Nature of behavior*	Direct experience/past behavior including routine, automatic, or habitual behavior

*From the initial set of 12 domains included in the TDF [47]

We used the TDF to develop our interview guide given its breadth, flexibility, and direct links to theory on factors known to affect decision-making and behavior [47]. We worked with physicians to identify who has to do what differently, in what context, and at what point in time to screen for this trial [50]. Thus, we designed questions to explore barriers and enablers to physicians screening patients to determine eligibility for an early-phase CAR T clinical trial. We developed one main question and several prompts for each of the 15 domains based on the existing guidance [44]. The interview guide was then pilot tested with physicians within the research team for clarity prior to recruitment (see Additional File 1 for the full interview guide).

### Recruitment

Saturation is an indicator of quality and trustworthiness in qualitative research and refers to when sufficient data has been collected such that no new themes emerge. We used the 10 + 3 rule [51] to determine our target sample size and gauge whether we had collected enough data to delineate our domain subthemes. We sought to interview hematologists from across Canada who had experience treating patients with hematological malignancies. We contacted hematologists known to the research team (convenience sampling) by email. Physicians who expressed an interest in our study were invited to participate in a semi-structured phone interview.

### Analysis

We used qualitative data analysis software, NVivo Pro version 11, to conduct a directed content analysis using the TDF as the guiding theoretical framework [44, 47, 48, 52]. Interviews were independently coded by SA and MF and later compared to resolve coding conflicts. Coding consensus was reached by choosing the coding strategy that satisfied both analysts, thereby ensuring no elements in the data were missed due to individual biases. Coded data excerpts were converted into belief statements (SA) and revised by a third analyst (GC). Belief statements were compared within and across domains to generate and distinguish between domain-specific subthemes [53]. To document the progression from data excerpt to subtheme, we created belief statement tables showcasing subthemes, belief statements, exemplary quotes, case counts (i.e., number of participants endorsing a belief), and total frequency counts (i.e., how many times a belief appeared in the data set) (see Additional File 2). We identified key domains according to the frequency of beliefs, presence of conflicting beliefs, and relevance to screening behaviors [44]. To aid with this process, we sought out opposing and marginal views within the data set.

A detailed summary of results (see Additional File 3) was shared with a patient partner (TH) who provided feedback regarding what aspects were most important from a patient perspective. Subthemes and belief statements were further compared to identify overlapping, or related, barriers and enablers across domains. Recurring themes that transcended domains were derived from the data and organized or clustered into global themes that captured key tensions relevant to trial recruitment [54].

### Ethics approval and consent to participate

This study received ethics approval from the Ottawa Health Science Network Research Ethics Board (approval #20170502-01H) and from the University of British Columbia - British Columbia Cancer Agency Research Ethics Board (approval #H17-01472). Written consent was obtained from all participants prior to being interviewed.

## Results

### Interviews

A female-identified research coordinator (SA) with a master’s degree in health systems management and expertise in qualitative methods conducted all phone interviews. SA was trained in using the Theoretical Domains Framework and received guidance and feedback from an expert in Theoretical Domains Framework methodology and behavior change theory (JP). When conducting interviews, SA introduced herself as a health services researcher and clarified that she did not possess a clinical background. Interviews lasted from 17 to 35 min (median = 25 min). All interviews were audio-recorded and later transcribed verbatim.

### Participants

In total, 20 physicians were contacted and invited to participate in this study. Three did not respond to the invitation. Two expressed interest but did not respond to follow-up emails and were not scheduled for an interview. The remaining 15 (9 men, 6 women) hematologists were interviewed over the phone. Participants reported residing across 6 provinces. Specifically, 7 participants were based in Ontario, 2 were from British Columbia, 2 were from Alberta, 2 were from Quebec, one was from Manitoba, and one was from Newfoundland. Physicians ranged in their reported time spent treating patients with hematological malignancies from 2 to 37 years (median = 12 years). Every physician reported treating patients with lymphoma, leukemia, or both. Four also treated myeloma and two treated other hematological malignancies. Every physician had treated patients who were eligible for bone marrow transplantation and had participated in a clinical trial as a site investigator. Thus, every participant was potentially positioned to engage in the specific recruitment activities under study.

### Identified barriers and enablers to screening

We identified eight relevant domains: Knowledge, Beliefs about Consequences, Goals, Intentions, Social and Professional Role and Identity, Environmental Context and Resources, Memory/Attention/Decision Processes, and Behavior Regulation. These domains were grouped into three global themes. *Cautious Hope* describes the physician’s views about CAR T cell therapy (domains: Knowledge, Beliefs about Consequences, Goals, Intentions). *Challenging Contexts* describes the material and cognitive resource scarcity that characterizes clinical trial recruitment (domains: Beliefs about Consequences, Memory/Attention/Decision Processes, Behavioral Regulation, and Environmental Context and Resources). *Variability in Perceived Roles and Screening Procedures* reflects the differences in existing processes for how clinics identify and screen patients for clinical trial participation (domain: Social and Professional Role and Identity). Global themes, key domains, subthemes, sample belief statements, and quotes are presented in Table 2. Data saturation was reached for all key domain subthemes and global themes. A comprehensive list of detailed belief statements and subthemes associated with each domain are provided in Additional Files 2 and 3.

**Table 2:** Global themes, key domains, subthemes, and example belief statements and quotes

Relevant domains	Subtheme	Sample belief statements^a^	Sample quotes
**Global theme 1: Cautious Hope**			
Knowledge	Knowledge gaps about CAR T cell therapy (barrier)	I would like to know more about toxicity, adverse events, and treatment efficacy. (9)	“So I’d have to think about that a little bit more, but I think a session on side effects and toxicities and complications and how to potentially manage them so that we can counsel patients properly would probably be an important thing to include in a site initiation visit.” - *Physician #9*
	Knowledge gaps about screening procedures (barrier)	I need to know clear eligibility criteria to properly screen for the trial. (9)	“So you need clear eligibility so you know exactly what you’re screening for what the yes’s and no’s are and you’re not wasting anyone’s time in creating false hope… so I think it’s clarity of exactly what is required for the study.” - *Physician #12*
	Knowledge about immunotherapy trials (enabler)	I am aware of immunotherapy clinical trials for hematological malignancies. (13)	“…there has been approval for CAR T-cell products for B ALL in paediatric patients as well as another CAR T-cell product that’s been approved for aggressive lymphoma. And they’re currently, you know, trying to wrap up to try to make this more available generally speaking outside of clinical trials. ... And people are also trying to develop CAR T-cells outside of the realm of CD-19 antigen therapy so that should actually lead to other availabilities of different CAR T-cell products.” - *Physician #3*
	Information Delivery (enabler)	I would like to learn about CAR T in person (e.g., rounds, site visits). (5)	“I think presenting the background at our rounds or something is the useful thing.” - *Physician #8*
Goals	Concern for Patients (enabler)	CAR T trials are important because they provide patients with more treatment options. (8)	“But more importantly it gives patients an opportunity to access medications and treatments that may not be available for 5, 10, 15, 20 years. So I think it’s really important to put patients on clinical trials and especially for CAR T because… there’s a huge medical need for patients like that that we would actually think of for CAR T. There’s really nothing we can offer those patients.” - *Physician #2*
	Advancing Science (enabler)	Screening for a safety study is important to generate safety data. (4)	“I think there’s still a lot to be learned about it improving both the agent itself and improving management of toxicity. So I would be delighted to contribute to that learning because I do think it’s a big part of the future.” - *Physician #12*
	Support for Trial (enabler)	Screening for this trial is a priority. (6)	“Well given sort of this is the gold at the end of the rainbow I think we’d still be motivated to get involved.” - *Physician #14*
	Other motivators (enabler)	Trials with inclusive criteria motivate me to participate (1)	“Well easily accessible trials would motivate me because sometimes I do get quite discouraged with certain trials that even though the trials might be open but they have really stringent criteria and even if we screen the patient on an initial screen then they get a second screen and they are rejected. And that just leaves me and my patient with a lot of questions. So an easily accessible trial would really motivate me to enrol my patients because I know that they will make it in.” - *Physician #5*
Beliefs about Consequences	Treatment Benefits (enabler)	This trial presents a possible treatment avenue for patients with very limited options. (12)	“But more importantly it gives patients an opportunity to access medications and treatments that may not be available for 5, 10, 15, 20 years. So I think it’s really important to put patients on clinical trials and especially for CAR T because we just don’t have there’s a huge medical need for patients like that that we would actually think of as CAR T for. There’s really nothing we can offer those patients.” - *Physician #2*
	Advancing Science (enabler)	This trial will add to the CAR T knowledge base. (4)	“Well obviously the, the benefit is that one adds to the literature, adds to the knowledge on how certain treatments work. So the benefit is that of any research effort is research result.” - *Physician #7*
	Toxicity (barrier)	CAR T early trials are likely to have high toxicity and mortality rates. (10)	“Well you’d be going through the potential benefits of CAR T-cell therapy also the risks of it and the risks obviously are quite significant with cytokine release syndrome etc.” - *Physician #11*
	Insufficient Evidence (barrier)	There is not enough data regarding side effects and efficacy. (3)	“We’re only beginning to understand it right now. We don’t know where it’s going to be heading in the next 4-5 years. The early results look really promising but for every patient out there who’s benefited from CAR T therapy there’s a patient conversely who hasn’t benefited from it. And so there’s still lots to learn about it so the disadvantage is that we just don’t have enough information about the long-term outcomes of these patients whether patients are going to be cured of their diseases or not. So there are so many disadvantages that there’s a lack of knowledge right now about the long-term.” - *Physician #2*
	Financial Cost (barrier)	It is costly to administer CAR T therapy and manage complications. (5)	“Many of them end up in the Intensive Care Unit making it quite an expensive treatment, but at the same time I think it carries along a significant survival benefit for the patients.” - *Physician #13*
	Feasibility and Screening Criteria (enabler)	The study design and feasibility are important to ensure enough patients are eligible. (7)	“The most important factors [are] a well-designed study, clear eligibility criteria and process for trial conduct. And I guess it also needs to be manageable. … if there were so many tests with inadequate funding to support then that might be something my institution would say this is too expensive. This is too intensive we cannot do it so a feasible screening process and well-designed study would influence me to screen.” - *Physician #12*
	Few Participants (barrier)	Few patients will be eligible for this trial. (5)	“We generally do need to be able to say from a feasibility perspective that we think we can enrol at least 3 patients onto a study otherwise the workload of opening it is felt not to be worth it for just a very small number of patients.” - *Physician #6*
Intention	Intent to Screen (enabler)	I intend to screen patients for the trial. (14)	“Yeah we’ll be willing and I think it’s not only a matter of choice it’s a need. We need that, it looks like this therapy is quite promising and has potential and most of these patients are in a desperate situation and this is actually, here in Canada, it’s a need. So it’s needed and I think myself, my colleagues, would be happy to have a trial and take part in this and to put subjects first.” - *Physician #4*
		I intend to screen patients for a safety trial. (7)	“Early phase studies here at our centre are harder to conduct ... Well given sort of this is the gold at the end of the rainbow I think we’d still be motivated to get involved.” - *Physician #14*
	Conditional Intent (barriers)	I would consider whether the patient could commit to the trial. (1)	“If they are not a compliant patient for the study schedule then I will not to include a patient to trial… I rather this person this subject is traded for person who can commit himself or herself to the study protocol.” - *Physician #10*
**Global theme 2: Challenging Contexts**			
Environmental Context and Resources	Institutional Capacity (enabler)	My centre has the resources to support a trial. (6)	“So we don’t hit too many hurdles here ourselves and in general if we think we have a good study that we can recruit patients to they [REB] will support us in making that work.” - *Physician #12*
	Access to Funding (enabler)	Funding is important to keep the study moving forward. (9)	“…it also has to be economically feasible for our clinical trials unit… the clinical trials group has to be responsible in trying to introduce studies that are not gonna drain the budgets for everybody else.” - *Physician #8*
	Access to Study Personnel (enabler)	Research personnel are needed for a smooth screening process. (8)	“… you have to have a number of research people who are working with you because they’re the ones who need to collect the data. They’re the ones who need to, you know, follow the patients with the physicians and that kind of stuff. So if you have a good research staff then it certainly makes life a lot easier for the physician.” - *Physician #3*
		Our medical staff/clinical trial team is able to screen. (4)	“Yeah, yeah and I know most of the, we’re kind of lucky all of our staff has some medical training and so they would actually go through the screening process in detail.” - *Physician #2*
	Physician Time and Workload (barrier)	Screening requires a lot of work and resources. (6)	“You can call somebody and say come on down and talk to this patient and look at the chart and see if it fits—the typical study is like that and it would be like this too I think although this is so complex this, this therapy and so expensive and so unusual that one would—this is the it becomes a project.” - *Physician #7*
	Trial Availability (barrier)	We avoid having competing trials. (8)	“We try not to. So that it’s so we wouldn’t be able to run competing studies for especially in this disease.” - *Physician #8*
Beliefs about Consequences	Wait Times (barrier)	Long wait times for screening and time to treatment may be difficult for patients with aggressive diseases. (8)	“I’m not sure what is the turnaround time for the development of this technology specific for that patient, so the patient could die or deteriorate in that time period. And you have to remember these are people, so say if we are only enrolling people who have multiply relapsed refractory disease then I think it might be a case where the patients are really sick. So I think that might be one of the disadvantages.” - *Physician #5*
Memory, Attention and Decision Making	Forgetting to Screen (barrier)	It is possible to forget to screen for this trial. (5)	“If I’m too much busy or too many patients while I’m running the clinic there I may forget.” - *Physician #10*
		I will not forget to screen for this trial. (9)	“No way [laugh] it’s such an important trial, it would be at the forefront of my mind constantly so no I wouldn’t be concerned about that at all.” - *Physician #11*
	Patient Health (barrier)	I would need to consider their health status. (6)	“The only time I will think twice about offering them a clinical trial is based on their echo [echocardiogram] performance status, their clinical status, but even then I do mention it to them with a caveat that by the time they get enrolled it might be too late, yeah.” - *Physician #5*
	Treatment Options (barrier)	I would consider if other treatment options are available. (7)	“Because if I have some other therapies to offer them to get them better, rather than sit and do nothing for 2 weeks until I get an answer whether they’ll be enrolled in the trial or not, then I might decide not to enrol them in the trial.” - *Physician #5*
Behavioural Regulation	Salience of Trial Improves Screening Practices (enabler)	Sending reminders to physicians helps keep screening on track. (6)	“So reminders are very helpful, any kind, that every time we have someone come by and give us a reminder on our clinical trial it tends to improve accrual. So, you know, email reminders or, you know, just a small update on accrual, newsletters, all those things that people use for trials I think are helpful in terms of reminding us.” - *Physician #13*
		Screening patient charts on a regular basis may help us identify patients for a clinical trial (5)	“so there are two ways, one is we meet every week to discuss patients and their care to make sure everything is moving on track and whether there are options for certain patients. And, I think presenting the background at our rounds or something is the useful thing.” - *Physician #8*
		Having committed hematologists to screen for the study will help. (2)	“…heightened awareness by the group rather than and, of course, you have champions for studies such as [this], like there’s the PI and keeps reminding people.” - *Physician #7*
		Involving non-profit organizations can increase exposure and salience. (1)	“There’s a place called CARE or an organization in [city]. And they basically give you the updates of all the trials and they also give you information ... they give it usually to hematologists. So that would be some good places to start right there.” - *Physician #6*
**Global theme 3: Variability in Perceived Roles and Screening Procedures**			
Social and Professional Role	Perceptions of Screening Role (enabler)	It is a physician's responsibility to screen patients for a trial. (15)	“It’s the physician’s responsibility to evaluate patients for a clinical trial if there’s a change in their disease status, right. So, if a patient comes in and they’ve relapsed it’s up to the patient’s physician to say okay what clinical trials do I have for this person. Are they eligible and if they are if they do look eligible then to start the process going to get them enrolled.” - *Physician #1*
	Variable Division of Responsibility (barrier/enabler)	Screening is done by the physician, clinical research coordinator and medical staff. (13)	“Yeah I think, I think with likely the research nurse who would do the actual screening but, you know, hopefully patients would be, would be referred to the hematologist for consideration and assessment and then, you know, in, in turn the research nurse would become involved in sort of scrutinizing the inclusion and exclusion criteria and establishing candidacy. But I think it would be sort of a combined, you know, collaborative effort between those 2 people.” - *Physician #15*
		It is a shared role amongst physicians to screen patients for a trial. (6)	“And all patients of a certain type will go to one group of physicians. In our institution right now we all see a little bit of everything and that may change but that does mean as a group of clinicians here in [city] sometimes we’re perhaps less thorough at screening than we could be because it’s diluted across multiple physicians.” - *Physician #12*
		I pre-screen. Full screening is usually done by the study PI or Co-PI. (5)	“But if you are not on-service and you don’t know about this patient then it’s, it’s what they tell you and what the for example if you are the expert in a certain type of diseases in a group then it’s up to the other colleagues to ask your opinion and ask if this patient is a potential candidate or not. So to affect this process in the sense that you can’t know all patients in a big program it’s up to other physicians also to be motivated and take part in the trial or at least refer patients to the clinical trial.” - *Physician #4*
	Information Provider Role (enabler)	I provide patients with enough information to make an informed decision. (5)	“So for example if it’s my patient then I’ll do the initial screening like I said and then they would go to the clinical research team. And the research team would evaluate things and then go through the patient one more time. And if the patient actually consents to the study, then I would come in and I would try and answer more of their questions. And even if they haven’t consented to the study when they’re thinking about it then I’ll still go back and talk to them about it. The idea being to give them as much information as possible as they need in order to make a decision one way or another.” - *Physician #2*

^a^Numbers in brackets indicate how many participants endorsed a specific belief statement

#### Cautious hope

Physician perceptions of the planned CAR T cell therapy trial were cautiously hopeful. Most (*n* = 12) believed a CAR T cell therapy trial would benefit their patients and that CAR T cell therapy would fill a significant treatment gap (domain: Beliefs about Consequences). Nevertheless, hematologists were concerned about the side effects (*n* = 10) (domain: Beliefs about Consequences) and indicated a desire to know more about response rates, toxicity, and how to manage adverse events (*n* = 9) (domains: Beliefs about Consequences, Knowledge).

Many (*n* = 7) suggested that study design, feasibility, and inclusive eligibility criteria would be important factors when considering whether to screen potential participants (domain: Beliefs about Consequences). One physician indicated that the time investment needed to participate in a CAR T trial may not be worth the expected three to four patients that may be eligible to participate (domain: Beliefs about Consequences). Another physician indicated they would be more motivated to screen for the trial if they perceived the inclusion criteria to be sufficiently broad (domain: Goals). Despite these hesitations, 14 out of 15 physicians indicated they intended to screen patients for the upcoming CAR T cell therapy trial (domain: Intentions). However, certain factors might impact their intention to screen including the feasibility of travel, preference for efficacy over safety trials, availability of other treatments, and whether they believed a patient would be “a good fit” for the trial (domain: Intentions).

#### Challenging contexts

Several physicians (*n* = 9*)* noted that participating sites may require funding to cover the costs of tests, biopsies, medications, coordination of care, hospital beds, and time in intensive care units (domain: Environmental Context and Resources). However, they suggested that well-resourced hospitals (e.g., access to clinical trials team) may require fewer supports (*n* = 6) (domain: Environmental Context and Resources).

Further highlighting differences between sites, eight physicians specifically mentioned the need to fund additional research staff, while four indicated that their medical staff members were sufficiently trained and able to complete screening activities. Others (*n* = 4) indicated that covering patient travel costs would be an important consideration when screening. One physician suggested caregiver expenses should also be covered (domain: Environmental Context and Resources).

Several hematologists (*n* = 8) expressed concern regarding how the anticipated time delay between identification and CAR T cell infusion might impact eligible patients facing a rapidly progressing disease (domain: Beliefs about Consequences). Working in this high-pressure and time-sensitive setting, some (*n* = 5) suggested that they may forget to screen for the trial during a busy clinic or if there had been a long time-lapse between trial setup and screening (domain: Memory). Most physicians (*n* = 9), however, indicated they were unlikely to forget to screen patients given the importance of the trial (domain: Memory). Physicians additionally offered suggestions for increasing the salience of the trial and screening criteria. Proposed strategies included electronic prompts and reminders (*n* = 6), screening patient charts on a regular basis (*n* = 5), having a study champion to oversee recruitment (*n* = 2), and involving nonprofit organizations to increase exposure (*n* = 1) (domain: Behavioral Regulation).

#### Variability in perceived roles and screening procedures

Participants unanimously (*n* = 15) agreed that eligibility screening was an important part of their role given that cancer clinical trials are used as both first-line and last resort treatments. When describing the recruitment practices of their specific clinical setting, many indicated that their role varied from identifying to pre-screening, full screening, and to obtaining consent from patients. Some (*n* = 6) indicated that identifying and screening patients was a shared role among physicians. Some believed the shared approach to patient care ensured no eligible patients were missed during the screening process. However, one physician believed that the shared responsibility sometimes led to less rigorous screening procedures. Others (*n* = 5) suggested that as attending physicians, they would identify patients, pre-screen for eligibility, and then refer the patient to the study investigators who would carry out the formal screening process (domain: Social and Professional Role). One participant suggested that attending physicians then have to be motivated to screen for another investigator (domain: Social and Professional Role).

Hematologists also differed on their perceived role during the consent process. Some (*n* = 5) emphasized the importance of explaining procedure details, answering patient questions, meeting with family members, and ensuring they provided patients with sufficient information to make an informed decision regarding trial participation while others did not (domain: Social and Professional Role). Regardless, most physicians (*n* = 13) agreed that research staff helped to alleviate their workload. They described often working in tandem with the clinical research staff, who were often responsible for providing detailed study information, obtaining consent from patients, and coordinating other aspects of clinical trial participation (e.g., tests, samples, follow-ups) (domain: Social and Professional Role).

## Discussion

Hematologists in this study were highly motivated to screen for a CAR T cell therapy trial and wished to know more about efficacy and toxicity. Many indicated that sufficiently broad eligibility criteria were an important aspect of the study design. One physician shared that they would be less motivated to screen for a trial if they believed eligibility criteria were too narrow. Hematologists also identified several barriers to screening including limited resources, forgetting study details, and forgetting to screen for the trial. An unanticipated but important issue raised was the timing between identifying an eligible patient and the inherent delay required for manufacturing their CAR T cells. Finally, they described variability in screening roles and consent procedures suggesting that current models of care may hinder or support recruitment processes depending on site dynamics and available resources.

Very few studies have explicitly assessed barriers and enablers to trial participation in the context of hematological malignancies and from the perspective of hematologists. Only one study to our knowledge has identified recruitment challenges in this context. In a retrospective cohort study, Lemieux and colleagues found that only about 50% of patients with hematological malignancies who were potentially eligible for a trial were recruited [55]. The most common reasons for non-recruitment included failing to meet all the inclusion criteria, not being approached by their physician, and patient refusals. While the authors reported on patient reasons for refusals, no information was provided regarding physicians’ decision to abstain from presenting patients with a trial. Other studies have similarly found that physicians do not always approach eligible patients about participating in clinical trials [23, 25]. Our study adds to the knowledge base regarding barriers faced by hematologists when recruiting to early-phase clinical trials and suggests possible explanations for why physicians may not approach potentially eligible patients. We discuss three barriers—narrow eligibility criteria, forgetting trial details, and variable roles—that may impede the recruitment process for the CLIC-01 trial and other similar trials.

### Narrow eligibility criteria and safety concerns

Hematologists in this study suggested it would be important for eligibility criteria to be sufficiently broad. This echoes findings from other studies where strict eligibility criteria were identified as a barrier to early-phase clinical trial recruitment [13, 42, 56]. However, hematologists in this study also expressed concerns regarding the potential risks and uncertainties associated with CAR T cell therapy. Another study assessing prospective barriers to an early-phase stem cell trial for stroke similarly found safety concerns may prevent physicians from screening and recruiting patients to the early-phase trial [31]. Thus, physicians may experience a conflict between what motivates them to screen (broad eligibility criteria) and what they believe is necessary to ensure participant safety. This tension is apparent in the tendency to use strict eligibility criteria for early-phase trials [13, 42]. This suggests a balance must be struck between optimizing the breadth of eligibility criteria to enable feasible recruitment with careful consideration of patient safety.

A review of training programs for recruiters indicated that most training efforts have focused on communication skills, explaining complex concepts (e.g., equipoise, randomization), and employing shared decision-making frameworks [57]. We suggest training efforts include discussions regarding comparative data on efficacy, toxicity, and financial implications of CAR T cell therapy compared to similar therapies (e.g., bone marrow transplants, other CAR T cell therapies), competing trials (e.g., other immunotherapy trials), standard care (e.g., palliative care, chemotherapy), and no treatment. A rigorous approach to addressing physician concerns over safety as well as treatment costs, feasibility, and treatment efficacy will be to provide systematic review evidence of all available data on these factors [8]. Comparative evaluations may quell concerns regarding the efficacy and cost-effectiveness of CAR T cell therapy. However, while such foundational information may encourage physicians to screen for the trial, information provision alone is unlikely to optimize their recruitment activities given that recruiter training has not consistently improved recruitment rates [57]. Thus, considering the wider set of identified barriers to screening is necessary.

The Quintet Recruitment Intervention is one option that may be used to iteratively identify and address recruitment barriers alongside clinical trials [26, 27]. The Quintet Recruitment Intervention uses a combination of quantitative and qualitative methods to document existing recruitment processes and provide localized feedback to improve recruitment practices. The use of the Quintet Recruitment Intervention has helped identify and address barriers related to recruitment pathways and communication challenges [25, 27].

### Forgetting to screen amidst competing demands

Even the highly motivated hematologists in this study identified competing priorities and the potential for forgetting to screen as possible barriers to recruitment. Further, as recruitment extends over months, physicians indicated they may forget trial details that are provided during site initiation visits. The risk of forgetting trial details was identified in a survey study where 84% of physicians involved in clinical research at a cancer center reported difficulties keeping track of eligibility criteria for open trials [32]. However, studies evaluating the use of reminders for clinicians have not found a clear impact on recruitment rates [19]. Determining what aspects of interventions aimed at improving the salience of open trials are actually effective at improving recruitment rates is imperative.

Hematologists also suggested that workload may be a barrier to recruitment. One study found that decreasing physician workload was effective at improving recruitment rates [58]. Addressing workload concerns (clarifying from the outset the time commitment involved in terms of frequency of patients to screen) and simplifying screening and referral tasks may encourage better recruitment.

### Hematologist and oncologist roles in screening practices

To address the differences in site screening practices, it would be prudent to clarify “who does what” at each site and ensure that there is agreement on roles, especially when they differ from usual recruitment practices at a specific site. Identifying these differences early will ensure they are considered when trials become multi-center.

Of importance is clarifying physicians’ role in providing trial information. While physicians are not always involved in the consent process or information provision, many patients rely on their hematologists’ and oncologists’ assessments and professional opinions when making treatment decisions [59] (see also Castillo et al: Navigating choice in the face of uncertainty: Using a theory informed qualitative approach to identifying patient barriers and enablers to participating in an early phase Chimeric Antigen Receptor T cell therapy trial, forthcoming). A recent review similarly found that prospective trial participants appreciated hearing about trial information from individuals who were approachable, trustworthy, and knowledgeable about the trial and that healthcare providers were especially seen as trustworthy [16]. Considering both physician and patient perspectives is, thus, critical for optimizing recruitment to early-phase trials. It will be important for this, and future trials, to adopt strategies (peer support, improved access to specialists) that strive to meaningfully inform and engage patients while respecting physician workload and time [60, 61]. One recently evaluated strategy for addressing patient information needs while respecting physician time is the use of lay navigators [60]. In this study, non-physician staff were trained to provide informational, logistical, social, and emotional support to prospective trial participants. Participants receiving the lay navigation intervention were more likely to participate in a clinical trial [60]. If the use of lay navigators is not possible, developing materials that streamline physician time while providing consistent and accessible patient-centered messaging (e.g., videos of physicians describing the trial on a trial website, infographics for patients) may help address both patient and physician needs.

### Strengths and limitations

A possible limitation of this study is that it focused only on hematologists. Hematologists had been identified by the trial team as the most relevant healthcare provider group to interview in advance of the trial and were subsequently identified as important sources of trial information by patient participants. However, hematologists indicated that their screening and recruiting procedures often included other healthcare providers and research staff. As the evaluation of CAR T cell therapy moves to later-phase clinical trials and involves more sites, future research should also assess the barriers and enablers anticipated and experienced by other staff involved in the recruitment process.

The strength of this study lies in the use of the TDF to identify factors that may impact clinician screening and recruiting behaviors. The TDF enabled a comprehensive assessment of potential barriers and enablers to screening and recruiting to an early-phase trial of CAR T cell therapy. Qualitative methods further allowed for the exploration of anticipated as well as unanticipated topics to arise, such as the concern over wait times between screening and referring patients to the trial and patients receiving CAR T cell therapy. The TDF also enables linking research findings to theory in order to generate evidence-based strategies and, thus, provides a path for future research to build on the work presented here [49].

## Conclusion

This study demonstrates the value of using a comprehensive approach to identifying potential trial-specific barriers and enablers prior to conducting an early-phase cancer therapy trial to inform efforts to optimize recruitment activities. The methods (i.e., interviews based on the Theoretical Domains Framework) and tools (i.e., interview guide) described in this paper could serve as adaptable examples that can be used to inform recruitment activities in other future trials. Identifying and prioritizing barriers most relevant for a specific upcoming trial can guide what and how training and resources are used at trial launch and throughout to support physicians and other site staff in promoting consistent recruitment over time. As promising cancer therapies continue to develop at the bench, such approaches may help to optimize the conduct of trials so that effective therapies reach the bedside sooner rather than be delayed by trial recruitment failures.

## Supplementary Information

**Additional file 1:.** Physician interview guide.

**Additional file 2:.** Physician belief statements.

**Additional file 3:.** Physician subthemes.

## Data Availability

The full set of themes, categories, and belief statements supporting the conclusions of this article are available as additional files. Individual participant data will not be shared. However, we invite queries and comments from interested readers regarding the data or analytic process.

## Abbreviations

CAR T cell therapy: Chimeric antigen receptor T cell therapy
TDF: Theoretical Domains Framework
