# Supplementary material for: Hematologists’ barriers and enablers to screening and recruiting patients to a chimeric antigen receptor (CAR) T cell therapy trial: a theory-informed interview study
Source: Trials. 2021 Mar 25;22:230. doi: 10.1186/s13063-021-05121-y (PMC7995587; doi:10.1186/s13063-021-05121-y)
Supplement: Supplementary file 1 — Additional file 1:. Physician interview guide. [file 13063_2021_5121_MOESM1_ESM.docx]

**Supplemental Material 1 – Interview Guide**

**Target Action Context Time and Actor (TACT-A):**

Target: Patients

Action: Screening for a CAR-T cell therapy trial

Context: Hospitals, during clinics

Time: Before enrolling them in the trial

Actor: Hematologists

**Interview Guide for Physicians**

***Pre-amble***

- Thank you for making the time to speak with me about CD19 chimeric antigen receptor T-cell therapy, also known as CAR-T cell therapy, which may be a useful treatment for hematological malignancies. Our discussion should take approx. 30-45 minutes
- Audio-recorded to ensure key points are documented
- Any identifying info (e.g. name or names of others) used during our discussion will be removed from transcripts.
- If you want to end our discussion before I ask all questions or if you want to withdraw from the study you are free to do so.
- My background is in health services research and I’m not a clinician. I’m interested in your views about recruiting for CAR-T cell therapy trials and what might help or stop you from doing so. There are no right or wrong answers, and I’ll probably ask you for clarification throughout our discussion if that is ok!
- Your responses will be anonymized so that no one will know what your specific answers were.

Any questions before we start?

**Background of Informant**

- 1. Male or Female (to keep track of, will not be asked)
  2. How long have you been a physician caring for patients with hematological malignancies?
  3. Have you agreed to participate as a physician in a clinical trial previously?
  4. What diseases do you mainly treat?
  5. Do you see or treat patients who are eligible for stem cell therapy?
  6. Are you aware of any clinical trials using cellular immunotherapy for hematological malignancies?
  7. What do you know about how CAR-T cell therapies for leukemia and lymphoma?

For the duration of our discussion, I would like you to think of the following specific activity:

Patients who do not qualify for allogeneic stem cell transplant and have high risk disease or patients with relapse after allogeneic stem cell transplant might benefit from CAR-T cells, therefore throughout the next few questions, please consider a situation where you could **be the clinician screening patients diagnosed with CD19+ leukemia or lymphoma for participation in clinical trial for CAR-T cell therapy.**

Tell me about your past experience screening patients for a clinical trial.

- PROMPT: Have you screened patients for trials in the past? (*Nature of Behaviour*)
- PROMPT: (If yes) How did that go?
  - PROMPT: Have you ever received training to screen patients for a clinical trial as a clinician? (*Skills*)
- PROMPT: What sort of training would you need?

**General first question:**

Thinking about your own experiences or those of others, what are some of the barriers or situations that might make it less likely that you would personally be able screen patients in a trial of CAR-T cell therapy?

**Domain Knowledge (Constructs = Knowledge and Procedural Knowledge):**

What do you know about CAR-T cells in general, and for blood cancers in particular?

- PROMPT: Have you ever heard about CAR-T cells for treating blood cancers?
- PROMPT: Have any of your patients asked you about CAR-T cells?

1. *Knowledge:* What information would you need to feel comfortable screening patients in a trial of CAR-T cell therapy for hematological malignancies? What would be the best way to provide you with that information?
   - PROMPT: Brochure, website, smart phone app, meeting with trial team?
2. *Procedural Knowledge:* Imagine yourself screening a patient. Can you briefly walk me through the steps that you would use to go about that?

**Domain Skills (Construct = Skills):**

1. *Skills*: Have you received training in how to screen patients for heme malignancies previously?
2. What are the specific techniques or skills that you need to screen for enrollment for this trial?

**Domain Social/Professional Role and Identity (Construct = Professional Role):**

1. How do you see screening for this trial in relation to your role as a clinician?
   - PROMPT: Do you see this as something that is a part of your current role?
   - PROMPT: Do you think it should be part of your role?
   - PROMPT: (If no) Whose role should it be?
   - PROMPT: What would be your responsibilities in this role?

**Domain Beliefs About Capabilities (Constructs = Self-efficacy)**

1. You mentioned a few reasons that might stop you from being able to screen patients for a clinical trial. How confident are you that you could screen patients for a trial of CAR-T cell therapy for treating blood cancers?

- PROMPT: What might make you feel more confident?
- PROMPT: What might make you feel less confident?

**Domain Optimism (Constructs = Optimism and Pessimism)**

1. *Optimism:* Do you expect that your screening of patients in a trial with CAR-T cells for hematological malignancies will result in more good things than bad for your patients?

**Domain Beliefs About Consequences (Constructs = Attitudes and Outcome Expectancies):**

1. *Outcome Expectancies:* What are some of the benefits of screening for a clinical trial of CAR-T cells for hematological malignancies?
   - PROMPT: for patients enrolled in trial (e.g. reduce symptoms; what symptoms?)
   - PROMPT: for future CD19+ acute leukemia or lymphoma patients
   - PROMPT: for advancing research
2. *Outcome Expectancies:* What are some of the disadvantages that you see of screening patients for a trial of CAR-T cells to treat blood cancers?
   - Prompt: for patients enrolled in trial (risks for patients – what are the risks?)
3. **OFFER TO DESCRIBE POSSIBLE POSITIVE AND NEGATIVE CONSEUQNECES OF THE PATIENTS TAKING PART IN THIS TRIAL (EG SIDE EFFECTS, REMISSION). Once described, ask:** *Do the benefits outweigh the potential harms?*
   - Side effects:
     - Negative:
       - Cytokine-release syndrome (CRS), Tumour lysis syndrome (TLS), Neurologic toxicity, “on target/off tumour” recognition (B-cell aplasia), Anaphylaxis, graft v. host disease for allogenic cells
     - Positive:
       - Remission; increase in progression free survival; cure of cancer

**Domain Intentions (Construct = Intentions):**

1. *Intentions:* Would you screen your patients as part of a trial of CAR-T cells for hematological malignancies if one is available in the next year?
2. *Intentions:* Do you think your patients would be willing to participate in a trial of CAR-T cells for hematological malignancies if it impacted their ongoing treatment?
   - PROMPT: Why/why not? *How does this affect your intention to screen?*
3. How motivated would you be to screen patients for a trial of CAR-T cells if its goal was to test the safety of CAR-T cells, but not to test if it improved your patients’ health?

**Domain Goals (Constructs = Priority):**

1. *Priority:* Generally, in your clinical practice, how often might something else be a higher priority than screening for this clinical trial?
   - PROMPT: Might something be more urgent?
   - PROMT: How do you think this will impact your ability to screen?
2. Would it be easier to recruit/screen during clinical duty or when you are off service? (BelCAP)

**Domain Reinforcement (Construct = Reinforcement):**

1. *Reinforcement*: What would motivate you to screen patients to participate in a trial of CAR-T cell for hematological malignancies?
   - PROMPT: patient benefit, science, friendly competition in terms of recruiting compared to my other colleagues as to who recruits most/least; being acknowledged in the academic outputs from this trial?

**Domain Memory, Attention and Decision Processes (Constructs = Memory and Attention)**

1. *Memory:* Do you think it is likely that you might sometimes forget to screen for this trial? When would you most likely forget? What might help?

**Domain Behavioural Regulation (Constructs = Automaticity, Self-Monitoring, and Action Planning):**

1. What are some strategies you might use to stay on top of screening patients?
   - PROMPT: What can you do personally to help stay on top of screening patients?
   - PROMPT: Is there anything in the past that has helped you stay on top of screening patients that would help us to build into our processes for this trial?
2. *Nature of Behaviour:* To what extent would screening become part of your routine practice?

**Domain Emotion (Constructs = Affect and Stress):**

1. *Affect*: How do you think your feelings may influence you screening patients for a trial of CAR-T cells for hematological malignancies?
   - PROMPT: (prompt key potential emotions: guilt, worry, concern, satisfaction inspired, nervous, stressed, happy, sad)
   - **Tense situation, patient received bad news:** how would that influence your screening?
2. *Stress*: Would screening add any level of stress to your current workload?
   - PROMPT: How would that stress influence screening for this trial? (ie. Make reluctant, not want to get involved, etc.)

**Domain Social Influences (Constructs = Social Support, Subjective Norm, and Descriptive Norm):**

1. *Subjective Norm:* Who else’s views might have an impact on whether you decide to screen patients for this trial?
   - PROMPT: Patients, their family, other physicians, colleagues, your family (time)
2. Whose support would help you decide to screen patients for this trial? Prompt: Institutional support (hospital), or trial team assistance enrolling patients?

**Domain Environmental Context and Resources (Constructs = Resources Material):**

1. *Resources Material:* What resources do you need to be made available for you to be able to screen patients for this trial? And to what extent do you already have access to these resources?
   - Prompt: Financial
   - Prompt: Logistical challenges?
2. Is there anything about the physical setup of the unit/ward itself that (could) influence whether or not you screen patients?
   - Prompt: space, time and place to do this
3. What resources at your center do you feel would really facilitate your ongoing treatment of patients after a trial of CAR-T cells for hematological malignancies?

*Final question!:* Thinking about everything we’ve just discussed, in your opinion, what are the most important factors that would influence whether screen patients in a trial of CAR-T cells for your patients diagnosed with acute leukemia or lymphoma?

***Is there anything else you wanted to say or expand on about?***

***Thank you for participating.***
