# Supplementary material for: Hematologists’ barriers and enablers to screening and recruiting patients to a chimeric antigen receptor (CAR) T cell therapy trial: a theory-informed interview study
Source: Trials. 2021 Mar 25;22:230. doi: 10.1186/s13063-021-05121-y (PMC7995587; doi:10.1186/s13063-021-05121-y)
Supplement: Supplementary file 3 — Additional file 3:. Physician subthemes. [file 13063_2021_5121_MOESM3_ESM.docx]

**GO CART Hematologist Subthemes and Belief Statements**

**Most Relevant Domains**

The most relevant domains were identified based on the criteria outlined by Atkins et al. (2017). Relevant domains were chosen based on the frequency with which beliefs and themes appeared in each domain, the presence of conflicting beliefs, and perceived relevance to the target behaviour.

| **Domain** | **Number of participants represented** | **Total number of instances coded to domain** |
| --- | --- | --- |
| [Reinforcement](#_Reinforcement) | 2 | 2 |
| [Nature of behaviour](#_Nature_of_the) | 6 | 9 |
| [Beliefs about capabilities](#_Beliefs_about_Capabilities) | 10 | 16 |
| [Optimism](#_Optimism) | 12 | 18 |
| [Emotion](#_Emotion) | 13 | 24 |
| [Skills](#_Skills) | 12 | 25 |
| [Social influence](#_Social_Influence) | 14 | 27 |
| [**Behaviour regulation**](#_Behaviour_Regulation) | **10** | **33** |
| [**Memory, attention and decision making**](#_Memory,_Attention_and) | **14** | **41** |
| [**Intention**](#_Intention) | **15** | **43** |
| [**Goals**](#_Goals) | **15** | **58** |
| [**Social and professional role and identity**](#_Social/Professional_Role_and) | **15** | **86** |
| [**Beliefs about consequences**](#_Belief_about_Consequences) | **15** | **108** |
| [**Knowledge**](#_Knowledge) | **15** | **118** |
| [**Environmental context and resources**](#_Environmental_Context_and) | **15** | **132** |

# Environmental Context and Resources

***N* = 15**

***Material Resources***

Institutional Capacity

Several physicians (*n* = 11) discussed how institutional capacity impacted their ability to screen patients. For hematologists that worked in academic hospitals, they perceived few environmental and contextual barriers (*n* = 6) suggesting that little about their unit or institutional set up (*n* = 5) prevented them from screening patients:

I: would anything about the physical set up of the unit or the area impact in whether, you know, it makes it easier for you to screen or not?

R: Well no, no the screening is well the screening is most often done in the clinic I think and in discussions and behind closed doors. The ward would have to be set up appropriately, but it probably is already more or less, yeah.

*Physician #7*

***

R: So, we don’t hit too many hurdles here ourselves and in general if we think we have a good study that we can recruit patients to they [REB] will support us in making that work.

*Physician #12*

Six physicians noted, however, that smaller hospitals may not have the resources to support a CAR-T, early phase, or investigator-initiated trials. They suggested that smaller institutions may not have the infrastructure and expertise to administer CAR-T cells or that they would need additional funding to ensure they could secure the resources needed to run an early phase, investigator-initiated CAR-T clinical trial.

R: Now the other thing with CAR T would be how can we have the infrastructure to, to make the CAR T therapy. So, for example if we lacked the technology or the facilities to make CAR T then more likely we will say we can’t participate because we just don’t have that infrastructure.

*Physician #5*

***

R: And we also need to make sure that we have the infrastructure that the trial needs. So, for example some might need lab you know just simple CBC and diff. Other trials may need to do stem cell collection. So those are 2 very, very different things and we want to make sure that we have the resources to meet the needs of the clinical trial.

*Physician #1*

***

R: Early phase studies here at our centre are harder to conduct because often they often involve more drug level monitoring, more PK studies.

*Physician #14*

Funding

A related concern voiced by 12 hematologists had to do with available funding to support the trial. Nine physicians mentioned the need to provide trial sites with funding to cover the costs of a variety of procedures including blood and imaging tests, biopsies, trial related treatments, coordination of care with other departments, hospital beds, the costs associated with the intensive care units in case of adverse events, as well as administrative aspects of the trial like REB approval.

R: But some of the things you may be able to impact would for example be funding for centres because I know across centres everyone’s different in the amount of resource they allocate. And a lot of centres have to be able to cover all costs of clinical trials. So, I’m not sure if you’re considering start-up funding or if you’re considering what your estimated per case funding is. But being sure sites are numerically and financially reimbursed for the workload is helpful to pay for clinical trial staff and extra radiology tests and whatnot.

*Physician #12*

Seven doctors specifically mentioned the need to fund research personnel and four also mentioned the need to provide trial participants with resources and support, especially when they are required to travel to treatment sites. One physician also highlighted the need to consider caregiver needs. Another physician mentioned that compensating hematologists for their time was not necessary.

R: I guess one of the most important things is on a practical level you have to have the resources, the financial [support] to be able to do this. The difficulty with clinical trials is that they require a lot of trial support in terms of personnel to help out with this. So, a well-funded clinical trial always gets done better than one, of a clinical trial that’s not well-funded. So, on a practical level you have to have the money backing it.

*Physician #3*

***

R: So, if a study I mean you need to really think about everything that patient needs to do, you know, including, you know, make sure that they got phone cards so they can phone back home. I mean you could be away for a while, right, and, you know, these patients are away during the sickest time in their lives and maybe the most stressful. So, you need to really think about everything the patient and, and a caregiver needs. I wouldn’t actually send someone away on their own I’d say that they should they absolutely need to have a caregiver and they need to have a caregiver plan if there’s burnout or if you need like, you know, tag-team caregivers those kind of things, right?

*Physician #6*

***Human Resources***

Access to Study Personnel

The need for study personnel was mentioned multiple times (*n* = 8). In addition to those who suggested their centre would need funding to compensate research staff, others suggested that trials must have designated research staff for proper screening, coordination, and data collection. Many physicians shared that they often worked with clinical research nurses or research coordinators during the screening process and that this professional support helped to ensure a smooth recruitment process and to lighten the screening workload. One doctor suggested that language barriers may prevent proper screening if medical staff are unable to properly explain CAR-T procedures to prospective participants.

Because I guess, you know, the truth is that, you know, you have, you have to have a number of research people who are working with you because they need they’re the ones who need to collect the data. They’re the ones who need to, you know, follow the patients with the physicians and that kind of stuff. So, if you have a good research staff then it certainly makes life a lot easier for the physician.

*Physician #3*

***

Language barrier is an issue for any trial not just specifically for CAR T-cell therapy. But because it’s a technology that requires some amount of understanding of what, what will happen and what are the potential side effects and complications that tends to be an issue. And I think, I think like any therapy that you may not necessarily have a lot of experience with or may not know a lot about it’s difficult to screen and to obtain proper consent if you feel you’re unable to, unable to communicate some of the, some of the certain nitty-gritty details of, of the therapy particulars to patients. So, I think that may potentially be a barrier for any clinician who doesn’t have particular experience in that area.

*Physician #9*

However, depending on available resources, not all sites will require designated staff. Four physicians also indicated that their medical staff were trained and able to conduct trial screening without additional support.

R: Yeah, yeah and I know most of the, we’re kind of lucky all of our staff has some medical training and so they would actually go through the screening process in detail. *Physician #2*

Physician Time and Workload

One of the reasons physicians may have emphasized the need for study personnel is that many (*n* = 6) perceived trial screening as work intensive and time consuming. They noted that time is needed to explain and discuss clinical trials as a treatment option for patients who have recently relapsed or have otherwise been identified as potentially eligible. Following identification, participants must be screened for eligibility, sent for various tests, and then consented for study participation. Even with the help of nurses and study staff, this process takes a considerable amount of time.

R: It’s a lot of work... our clinics are set up so that we’ve got about, you know, 10-15 minutes for a follow-up and usually these patients come in for a follow-up visit and oops they’ve relapsed. And so suddenly a 15-minute visit turns into an hour-long festival of organizing tests and arranging investigations and considering what, what studies they might be eligible for. We don’t actually have, you know, once the patient’s test results are back we don’t actually have a clinic where, where these patients come. They would just get slotted into another 10-minute, 10-minute follow-up slot. And then you have to explain, you know, here’s how CAR T-cells work. Here’s how they might benefit you. Here’s some of the side effects. I think you’re eligible for this study let’s have the clinical study nurse come and, you know, she can tie up my examining room for another 45 minutes so that I can’t see any more patients. So that’s the type of barriers it’s more systematic than, you know, individual barriers I guess.

*Physician #1*

Despite the challenges illustrated above regarding screening during clinic hours, two physicians indicated they preferred screening during clinic hours and three suggested it did not matter when they screened for trials. Two physicians indicated that screening for trials did *not* significantly add to their workload.

R: … actually, I think it would decrease the workload because now we’re looking outside the country so.

*Physician #13*

***Context***

Trial Availability

Six hematologists indicated that one of the main barriers to screening patients for CAR-T has been the absence of available CAR-T trials. These physicians discussed how the affected patient population has few options and is need of new treatment options. Four physicians specifically noted that there are currently very few competing trials, so just by making a Canadian CAR-T trial available, a significant barrier will be overcome.

R: I think it’s, it’s not only a matter of choice it’s a need. We need that it looks like it’s therapy that is quite promising and has potential and most of these patients are in desperate situation and this is actually here in Canada it’s a need.

*Physician #4*

***

R: Well I mean whether we actually had access to a trial for CAR T-cells has been the main barrier.

*Physician #11*

***

R: There really isn’t much in terms of trials for relapsed refractory aggressive lymphoma or ALL so I think it should be this would be the winner.

*Physician #14*

On the other hand, four physicians believe CAR-T trials will become increasingly popular and that there may be competing trials in the near future. The degree to which this becomes a barrier will depend on trial sites and their policies regarding multiple trials. Several participants (*n* = 8) shared that their centres actively avoid competing trials. This suggests that if other CAR-T trials become available at the same time, special consideration should be taken regarding how sites decide to participate in trials. One doctor indicated that being faced with multiple trial options may pose a difficult ethical dilemma regarding best patient care practices.

R: But after the trial is, is open I think mainly the main issue is if there are any competing trials. It’s patient preference and background and physician motivation and having patients on clinical trials all this will affect the screening process.

*Physician #4*

***

I: Do you ever have competing studies looking for?

R: We try not to.

I: Okay

R: So that it’s so we wouldn’t be able to run competing studies for especially in this disease.

*Physician #8*

***

R: And well why would I do it here if I could do it there? Same cells, same [32:09] XXX as well, not the same cells but similar cells, right? So that’s why I think that would be probably the biggest challenge for this is how to ethically deal with a patient facing a decision to enter a study here versus making an application to the province to go out of the country.

*Physician #7*

# Knowledge

***N* = 15**

Knowledge of Trials

Most physicians (*n* = 13) were aware that CAR-T clinical trials were being conducted and that CAR-T technology continues to develop. Physicians expressed knowledge regarding different disease subtypes needing different CAR-T cells. They noted that the United States was more advanced in their research and development and that trials were already available in the US but that none were available in Canada for CD19 specifically.

R: Well I guess recently there has been approval for CAR T-cell products for B ALL in paediatric patients as well as another CAR T-cell product that’s been approved for aggressive lymphoma. And they’re currently, you know, trying to wrap up to try to make this more available generally speaking outside of clinical trials. There’s a lot of other research initiatives that are being done by separate companies affiliated with or linked with academic institutions. And over the next couple of years I would expect there would be more commercialization of these products. And people are also trying to develop CAR T-cells outside of the realm of CD-19 antigen therapy so that should actually lead to other availabilities of different CAR T-cell products.

*Physician #3*

Knowledge of CAR-T

When asked what they knew about CAR-T cell therapy, every physician had an understanding of CAR-T research findings and physiological mechanisms. Doctors emphasized different aspects of CAR-T cell therpay including efficacy rates, small sample sizes, known toxicities, genetic variations, general mechanisms, and associated costs.

R: Oh you know what I am learning is the best I can to try to understand it. I understand that it’s highly toxic and that that, every time somebody explains it to me I forget how it works to tell the truth. It’s basically manufacturing I think T-cells to using a virus or a Lente virus in order to infect certain cells like the CD-19 cell for ALL or probably a CD-20 cell for lymphomas. I think those are the 2 main diseases being treated by CAR T-cells now and infecting them and then when that happens there is a significant cytokine release in the patient. Many of them end up in the Intensive Care Unit making it quite an expensive treatment, but at the same time I think it carries along a significant survival benefit for the patients. I think that in the studies that have been done there are not a lot of big studies I think maybe small studies but studies so far are relatively small I think like in the range of 30 people or less. And those studies have shown promise, significant promise with I don’t I think it’s possibly 50% of people with ongoing response.

*Physician #13*

R: Sure yeah. So, I, I’m not sure that the whole story about their mechanism of action has actually been worked out. The CAR T-cells are, are T-cells that are autologous to the patient that are modified with a T-cell receptor that recognizes something on the surface of a malignant cell. And that distinguishes the malignant cell from the normal cell population in the patient. And the once the CAR T-cells are infused into the patient the theory goes that the modified receptor directs them to the cancer cell and the T-cells kill the cancer cells.

*Physician #1*

Only one physician indicated not knowing a lot about CAR-T cell therapy because it is not a relevant treatment option for their patient base. Despite their perceived lack of knowledge, they were able to identify the general mechanism associated with CAR-T cell therapy.

R: So, because most of the data I believe is in, in patients with leukemia which I don’t treat, I’m not aware of the, the literature in that area. But I do know that CAR T-therapy has come up quite in recognition because of some successful outcomes. And what I know about CAR T it is the primary antigen receptor therapy so they are directed against CD-19 on B-cell and at the same time they stimulate the T-cells. So, they are kind of doing the same thing.

*Physician #5*

Despite their considerable knowledge on the topic, several (*n* = 9) hematologists discussed wanting to know more about CAR-T toxicity, adverse events, managing side effects, treatment efficacy and study logistics.

I know about it but I need to know more details. And then I think that would be about it really. As long as I knew details of the procedure and, and what to tell patients I think that would be fine that’s all I would need really. I need to know details of not only the evidence but also the logistics so how it would actually work…

*Physician #6*

R: So, I’d have to think about that a little bit more, but I think, you know, a session, a session on side effects and toxicities and complications and how to potentially manage them so that we can counsel patients properly would probably be an important thing to include in, in a site initiation visit.

*Physician #9*

Screening Procedures

Most physicians (*n* = 13) indicated knowing about screening procedures and were readily able to describe the process their unit undertakes when identifying, screening and enrolling patients into a clinical trial. Physicians often discussed additional behaviours in their description of screening procedures including reviewing patient charts, identifying patients, obtaining pre-screening consent, ordering tests, assessing for eligibility, coordinating with study staff, educating patients and caregivers and obtaining informed consent for the actual trial.

R: Well I mean in every clinical trial you have inclusion criteria and exclusion criteria specified. So, they act as your guideline for your initial consideration of a patient for a trial. So, if a patient fulfils the inclusion criteria without fulfilling any of the exclusion then that’s the first step in the process. And then obviously you move on to actually approaching the patient and looking at specifics about the patient as to whether and the trial as to whether you feel it would be a good fit for that patient. And but generally the inclusion and exclusion criteria will cover most of those concerns or aspects that you may have or I may have. And then, then obviously the next step is to approach the patient and give them some education with regards to the trial you’re proposing they consider and take it from there.

*Physician #11*

This suggests that screening involves several actions and behaviours that may involve distinct barriers and enablers.

While three participants suggested that all they needed was the protocol to properly screen patients (“other than the protocol really we don’t need anything else”), other physicians (*n* = 9) indicated that it was important for them to have clear eligibility criteria and trial information.

R: So, you need clear eligibility so you know exactly what you’re screening for what the yes’s and no’s are and you’re not wasting anyone’s time in creating false hope. And so my NCI US experience was unfortunate because I knew, I knew that she was at 10.4 cm but in the list they sent me all it had was a minimum of 1.5 and the maximum of 10 was not there. So there was about a week of extra work and false hope on the patient’s part that could have been avoided if that was clear from the outset so I think it’s clarity of exactly what is required for the study.

*Physician #12*

***Educational Resources***

Nine doctors mentioned that they would like to receive information about the CAR-T clinical trial through various modes. Five participants mentioned wanting to receive information in person (e.g., site visits, meetings, rounds, conferences), three mentioned online resources, three indicated they would like ongoing access to the protocol and one shared a preference for written documents.

R: A rapidly, easily accessible website for the study. Something that doesn’t take a complicated password or login so that we can see rapidly have access in clinic all have computers I think or Smart phones to, to trial criteria.

*Physician #14*

Three physicians also mentioned the need for accessible educational resources for patients, including accessible consent forms.

R: The other thing that I’m going back to patients, patient information right now it’s one of the things I’m really aware of. When we write informed consent forms they’re ridiculous. They’re 18 pages sometimes which I don’t think is informed consent but that’s the world seems to be going. But I’m quite sure most patients and there’s literature to support it they often don’t get past page 3. So even some kind of tool I’ve seen in a couple of studies where there’s even just one pretty, one pretty coloured page with a flow chart or the key points that we could use in discussion with patients to highlight the most important aspects or they could take home just for the key things that are maybe important if they don’t get through an 18-page document. Something like that might be helpful.

*Physician #12*

# Belief about Consequences

***N* = 15**

***Perceived Benefits***

Physicians identified treatment benefits as the primary positive outcome of having patients participate in a CAR-T clinical trial. The majority (*n* = 12) indicated that a CAR-T trial is beneficial because it offers patients with a treatment option when they have few alternatives. Four suggested CAR-T cell therapy would be beneficial in general and three believed participating in the trial was a positive outcome, regardless of associated therapeutic benefits. Many voiced excitement and hope at the prospect of offering a cutting-edge therapy to their patients.

I think if you ask any cancer doctor, you potentially want to offer your patient something that could give them some benefit especially if they have a really bad situation. And, even if there is an ounce of hope I think that’s something that we would all consider a good possibility for patients who would fit that trial.

*Physician #9*

R: Hmm I don’t really think there are disadvantages to [laugh] to sort of like if you’re talking about like opening a study that provides options for patients the, you know, I, I it’s I don’t know what I would identify as a disadvantage. It’s, it’s an advantage for patients to have new and alternative therapies. It’s an advantage for us to be able to offer it. It’s an advantage for us to, to learn about it and it’s an advantage for us to, to be able to study it in the context of a clinical trial whether, whether it’s successful or it’s not successful.

*Physician #8*

As suggested by these two quotes, physicians expressed a lot of hope and excitement for CAR-T cell therapy. Their motivation was primarily focused on what is best for patients and specifically increasing their chances of survival.

A few physicians (*n* = 4) also suggested we stand to gain from studying CAR-T cell therapy and advancing the science and technology of cancer treatments.

R: Well obviously the, the benefit is that one adds to the literature, adds to the knowledge on how certain treatments work. So the benefit is that of any research effort is research result.

*Physician #7*

Two hematologists noted that having access to a CAR-T trial in Canada would reduce travel costs for eligible patients and one indicated that an indirect benefit to screening for a CAR-T trial would be learning more about a patient’s medical history.

***Perceived Costs***

While physicians mentioned anticipated benefits more often than they did costs, eleven physicians discussed their concerns regarding safety (*n* = 10), insufficient evidence regarding side effects and efficacy (*n* = 3), and unknown efficacy in general (*n* = 1).

R: This is a tough population to do safety studies in because, you know, you would probably be looking at every single adverse event and patients with relapsed leukemia for instance have lots of adverse events. Patients who’ve had a stem cell transplant have lots of adverse events so you’re probably actually looking that leads to a very large safety study if that’s gonna be your primary focus?

*Physician #1*

So, when you use the word screening. The screening patients is not a big deal one way or the other but actually putting patients on a CAR T clinical trial that does have disadvantages because CAR T’s a relatively new technology. We’re only beginning to understand it right now. We don’t know where it’s going to be heading in the next 4-5 years. The early results look really promising but for every patient out there who’s benefited from CAR T therapy there’s a patient conversely who hasn’t benefited from it. And so there’s still lots to learn about it so the disadvantage is that we just don’t have enough information about the long-term outcomes of these patients whether patients are going to be cured of their diseases or not. So there, there’s so many disadvantages that there’s a lack of knowledge right now about the long-term things related to these things.

*Physician #2*

One participant indicated that although side effects are likely, they believed there was enough information available to successfully manage toxicity:

So, I think, you know, again having no practical experience using this, this technology my understanding is the toxicities are, are reasonably manageable if you have sort of a protocol in place for, you know, for treatment of the cytokine release syndromes and, and the neurological stuff.

*Physician #14*

Although only one hematologist subscribed to this belief, how well toxicity and side effects are managed may potentially influence a physician’s screening assessment and decision regarding patient enrollment. It will be important to demonstrate to hematologists that toxicity management guidelines are evidenced-based and well-reasoned.

The cost of the treatment was also mentioned as cause for concern by five physicians. Interestingly, despite expressing cautions regarding efficacy, toxicity and financial costs, several participants noted the benefits at the same time.

R: there is a significant cytokine release in the patient. Many of them end up in the Intensive Care Unit making it quite an expensive treatment, but at the same time I think it carries along a significant survival benefit for the patients.

*Physician #13*

In comparing the anticipated costs and benefits that physicians associate with a CAR-T clinical trial, it becomes clear that every clinical team will have to assess treatment efficacy against toxicity when deciding whether to participant in the trial and whether a specific patient should be enrolled. It may be helpful to provide physicians with comparative data that outlines the toxicity, efficacy and treatment costs associated with standard care, CAR-T cell therapy, and no treatment, so that physicians can adjust their expectations and assumptions based on available evidence.

***Study Design and Feasibility***

Study design and feasibility was of great concern to at least eleven physicians. Many suggested that given the rarity of the CD 19 mutation, finding enough eligible participants to screen for the trial will likely be a challenge. In fact, five hematologists suggested they expect to identify very few eligible participants. Three suggested their site would not participate in the trial if they do not foresee being able to recruit sufficient participants, especially given the work involved in screening and monitoring trial participants.

R: so there is a feasibility of, you know, how a patient would we be able to enrol for this, right? So, so in ALL it’s in ALL it’s always an issue because we do have a, we do we have small population of ALL patients that we see in our, in our clinic that and that might be eligible for this so the, the number the local number would be, you know, might be small.

*Physician #8*

***

R: If the, if we all agree and we have the patient population and we have we can support the trial appropriately then we’ll go ahead with it.

*Physician #2*

Additionally, seven hematologists suggested that study design, broad criteria and the feasibility of the trial would influence physician’s motivation to screen potential participants. Given the described wait time concerns and perceived workload, one physician indicated that the time investment needed to participate in a CAR-T clinical trial may not be worth the expected 3-4 patients that may be eligible to participate.

R: Hmm the most important factors – a well designed study, clear eligibility criteria and process for trial conduct. And I guess it also needs to be manageable. If you were to come up with some ridiculous list and I’m sure you won’t but if there was so many tests with inadequate funding to support then that might be something my institution would say this is too expensive. This is too intensive we cannot do it. So, a feasible screening process and well-designed study would influence me to screen.

*Physician #12*

Others similarly indicated that trials with feasible and accessible inclusion criteria were preferred and would help with physician motivation.

R: Well easily accessible trials would motivate me because sometimes I do get quite discouraged with certain trials that even though the trials might be open but they have really stringent criteria and even if we screen the patient on an initial screen then they get a second screen and they are rejected. And that just leaves me and my patient in a lot of questions. So an easily accessible trial would really motivate me to, to, you know, enrol my patients because I know that they will make it in.

*Physician #5*

Wait Times

Time is a critical resource that affects all aspects of conducting a clinical trial. Hematologists discussed need to screen eligible patients and process CAR-T cells quickly given that those who are eligible for CAR-T cell therapy are often contending with a rapidly progressing disease. Eight physicians shared concerns regarding long wait times from the moment participants are identified to when they receive the CAR-T cell therapy.

R: And so, patients with a disease that’s very rapidly progressive may not actually survive long enough to have CAR T-cells generated for them. Similarly, patients with diseases that are in bad locations like the central nervous system may not do well long enough for CAR T-cells to actually be generated. So that would be a barrier to participation.

*Physician #1*

***

One of the other factors is time to accessing therapy. So, for example a patient might be eligible for a clinical trial but say by the time they are screened by the time they undergo all the requirement staging investigations and by the time they actually get to the drug it might be a little bit too late because say their disease is quite aggressive. So, I think that might also preclude patients from getting into the trial because if they need treatment now then they don’t have time to wait for, you know, trial screening etc.

*Physician #5*

# Social/Professional Role and Identity

***N* = 15**

Every participant in this study was involved in the process of screening prospective participants. One physician was also a clinical trial researcher. Doctors unanimously (*n* = 15) stated that screening for trials was part of their role, despite the noted workload associated with screening for trial eligibility. Most physicians believed it was their responsibility to screen patients for eligibility given that clinical trials are commonly used as last resort treatments for haematological cancer patients. Thus, although screening for clinical trials is not explicitly funded or considered standard care, several physicians include screening as part of their regular practice (*n* = 4).

R: I would say it would be a part of my role. I mean certainly there’d be more, you know, but really I think a lot of the standard of care for a lot of haematological malignancies is a trial so it should be trials are kind of part of what we do, right?

*Physician #6*

Participants in this study indicated that screening procedures involve a variety of medical professionals and that there are notable differences in screening processes across centres. In general, physicians described two main pathways to screening patients. One group (*n* = 5) suggested that, as attending physicians, they would identify patients, pre-screen for eligibility and then refer the patient to the study investigators (principal investigators, co-investigators, and sub-investigators) who would carry out the formal screening process in collaboration with research staff.

R: But, of course, we would likely have a couple of sub or co-investigators as well that would be trained and, and available to see patients as needed. But I imagine the majority of patients would go to whoever the local PI is?

*Physician #15*

Others (*n* = 6) indicated that identifying and screening patients was a shared role among physicians and residents, depending on the model of care adopted by the centre (e.g., team of physicians vs most responsible physician).

R: Well we’re, there’s maybe a core of us, core of 8 or 9 of us who treat haematologic malignancies and we have Tumour Boards every week so we are in contact about sick patients and we discuss best therapeutic options for patients. And so clinical trials come up and we usually discuss amongst ourselves and if the patient seems like they could be eligible for someone’s clinical trial they will be appropriately screened or referred to the study nurse.

*Physician #14*

However, one physician indicated that sharing screening responsibilities among multiple physicians was not always helpful and could sometimes lead to less rigorous screening procedures because the responsibility is diffused across multiple actors:

That and in our centre so far I know in some centres people divide and there’s 1 or 2 physicians do myeloma and 1 or 2 do leukemia so it’s all very divided. And all patients of a certain type will go to one group of physicians. In our institution right now we all see a little bit of everything and that may change but that does mean as a group of clinicians here in [city] sometimes we’re perhaps less thorough at screening than we could be because it’s diluted across multiple physicians.

*Physician #12*

This participant believed that access to research staff could improve screening processes as indicated by the quote above.

Most physicians (*n* = 13) described in tandem with the clinical research team (research coordinators, nurse practitioner, clinical trial staff, etc.) whereby the attending physician would be responsible for identifying, pre-screening, discussing options with patients, and overseeing the formal screening process, while the research staff would be responsible for providing detailed study information, obtaining consent from patients and coordinating other aspects of clinical trial participation (e.g., tests, samples, follow-ups), and sometimes engaging in screening procedures.

R: Yeah I think, I think with likely the research nurse who would do the actual screening but, you know, hopefully patients would be, would be referred to the haematologist for consideration and assessment and then, you know, in, in turn the research nurse would become involved in sort of scrutinizing the inclusion and exclusion criteria and establishing candidacy. But I think it would be sort of a combined, you know, collaborative effort between those 2 people.

*Physician #15*

it’s having a whole team because there are so many opportunities and so many competing priorities as you point out if you leave it to the one physician to do everything it won’t happen most of the time. Whereas if you could involve the clinical trials team perhaps the pharmacy team then that’s really helpful.

*Physician #12*

Knowledge of the coordination aspect of clinical trial participation is likely why research staff were highly valued, and many physicians indicated needing designated research staff and/or funding for study staff.

Finally, many hematologists (*n* = 5) saw their role as providers of critical information and emphasized the importance of explaining details about the procedure, answering any patient questions, meeting with family members and ensuring they provide patients with sufficient information to make an informed decision regarding trial participation. Most physicians also indicated, however, that informed consent is typically obtained by someone else.

R: So for example if it’s my patient then I’ll do the initial screening like I said and then they would go to the clinical research team. And the research team would evaluate things and then go through the patient one more time. And if the patient actually consents to the study, then I would come in and I would try and answer more of their questions. And even if they haven’t consented to the study when they’re thinking about it then I’ll still go back and talk to them about it. The idea being to give them as much information as possible as they need in order to make a decision one way or another.

*Physician #2*

This finding is notable given that many patients regard their attending physicians as expert knowledge holders and rely on their doctors’ assessments and professional opinions when making treatment decisions. The role of doctors and information provision is one that needs further exploration given that providing study details and obtaining informed consent are often done by research staff.

Taken together, these findings echo what other researchers have documented, namely that physicians are positioned as the gatekeepers to clinical trial participation as they are tasked with identifying, screening and informing patients regarding their treatment options and associated outcomes. Doctors have considerable influence at every step of the screening process and may be vulnerable to biases and errors. To ensure optimal recruitment, efforts should be made to ensure that study criteria are well known and understood by all physicians who may be tasked with any of the above-mentioned activities. It will also be worthwhile to address any physician questions and concerns regarding the efficacy, toxicity and management of CAR-T cell therapy so that patients are given the most accurate and balanced information possible. Future research should explore how to best inform patients while respecting physician workload and time.

# Goals

***N* = 15**

Physicians were primarily motivated by their concern for patients. Every hematologist expressed a preference for screening for trials that would benefit their patients. Eight participants indicated they were motivated to screen for a CAR-T clinical trial because it offered their patients an opportunity for treatment where no other options exist.

R: Well it depends on what context. I mean if, you know, if we have patients or if I have a patient that I feel would benefit from the trial that would become a priority for me because obviously they’re, you know, patients at that stage in the day with multiply relapsed leukemia you need to be thinking ahead and planning ahead to give them the best chance of survival ultimately.

*Physician #11*

Seven shared that they routinely enroll their patients in clinical trials because that is the best way to offer treatment:

R: Oh, absolutely because we, we do so many clinical trials and, and as I mentioned I’m we I try to put everyone on a clinical trial because I think it’s the best way to help patients.

*Physician #2*

Two others suggested they would prioritize a later phase trial with evidence for efficacy over a safety trial.

And, of course, you would probably be more enthusiastic about enrolling patients into Phase 2 or 3 trials where there is documented efficacy and the safety details have already been sorted out.

*Physician #9*

A few physicians (*n* = 6) were concerned with advancing the science of CAR-T cell therapy and indicated that screening for a CAR-T clinical trial was important because it added to their knowledge base and because safety data is an important first toward making CAR-T cell therapy widely available.

I think there’s still a lot to be learned about it improving both the, the agent itself and improving management of toxicity. So, I would be delighted to contribute to that learning because I do think it’s a big part of the future.

*Physician #12*

Half (*n* = 7) expressed general support for a CAR-T trial suggesting it would be a priority for them to screen for the trial, provided their centre has agreed to participate. Two physicians were enthusiastic about accessing Canadian produced CAR-T cells.

# Intention

***N* = 15**

When asked whether they intended to screen patients for the upcoming CAR-T trial, all indicated that they would. Those that said they would cited reasons such as wanting to offer their patients with treatment options when no others exist and wanting to contribute to the evolving science and technology for cancer treatments.

R: I would yeah like if study’s an option that is available then, then why not?

*Physician #5*

In total, seven doctors said they would screen for a safety trial because they recognized that safety trials are necessary, that they are often the only available option, and that for the right patient, it may be beneficial.

So even though we know that the focus would be on safety as opposed to efficacy I think for the right patient and for the right patient population that would be that would certainly be a possibility.

*Physician #9*

Six physicians indicated that certain factors might impact their intention to screen including feasibility of travel, preference for efficacy over safety trials, and whether they believed that patients stood to benefit from the trial:

I think you also need to believe in the study. So you need to as a physician if I think either that there’s a real chance this patient may benefit or that even if they don’t benefit and sometimes that’s the case in the early phase studies, you are making it clear to patients this is altruistic. You may not benefit from this but we may learn from your experience to help others in the future.

*Physician #12*

# Memory, Attention and Decision Making

***N* = 14**

Memory

Participants were asked whether any factors in their environment might cause them to forget to screen patients or forget eligibility criteria. While most responded that they were unlikely to forget (*n* = 9) given the importance of the trial, a few suggested forgetting may happen during a busy clinic (*n* = 5) especially if there had been long time lapse between hearing about the trial and screening for it. Given the concerns over recruitment feasibility, ensuring no patient is missed and forgotten will be an important strategy for the success of the trial. Physicians offered some suggestions for increasing the salience of the trial and screening criteria in the Behaviour Regulation domain.

R: Do I think it’s likely that I might forget to screen for it? No way [laugh] it’s such an important trial there’s no it would be at the forefront of my mind constantly so, no I wouldn’t be concerned about that at all.

*Physician #11*

R: If I’m too much busy or too many patients while I’m running the clinic there I may forget.

*Physician #10*

Decision Making

When discussing the factors that might impact their decision to screen, physicians indicated they would consider patient health (*n* = 6). Physicians discussed considering specific indicators of health like organ function and echocardiograms to determine whether patients were healthy enough to screen for an early phase trial.

R: So, you know, they have to be basically fit enough to go into a clinical trial. So they have to show really good performance status and the day after show really good organ function. And they also need to fit into the various criteria and disease stage etc. etc.

*Physician #10*

They also discussed needing to consider whether alternative treatment options were available (*n* = 7), especially in relation to their health status and urgency for action.

But somebody who’s admitted to hospital they might be much more sicker so even if a clinical trial is open you may want to think twice whether that patient is actually gonna make it to the clinical trial. Because if I have some other therapies to offer them to get them better rather than sit and do nothing for 2 weeks until I get an answer whether they’ll be enrolled in the trial or not, then I might decide not to enrol them in the trial.

*Physician #5*

One participant suggested they would also consider a patient’s state of mind and risk-taking behaviour.

R: You know it’s not that, it’s not that approval or that simple but on the whole you do need to be able to sit with a patient to see what their situation is and what their, their state of mind is, right. Patients are much greater risk takers than are doctors. So, they’re most of the time willing to take risks even if you’re not measuring the benefit, right.

*Physician #7*

# Behaviour Regulation

***N* = 10**

Doctors suggested they use a variety of strategies to improve participant accrual including unit level strategies like recruiting outside of their own hospital (*n* = 3), having multiple physicians on the team work to identify and screen (*n* = 1), and individual level strategies like striving to discuss the possibility of trials with their patients early on (*n* = 2), and keeping up to date with the literature (*n* = 2). These strategies were thought to improve the timing of when a novel therapy is offered so that eligible patients may be identified with enough time to enrol administer CAR-T cell therapy.

R: If we don’t we advertise outside of our centre and because most of these CAR T-cell trials are being done in academic, large transplant centres, you know, we have a broad population base to whom we advertise these studies for and then we try to get people to send these patients early before they deteriorate such that they can meet the inclusion/exclusion criteria. And then we enrol them on studies.

*Physician #4*

So, I think one problem will be if they if it’s one issue may be is if you don’t bring it up early I think the patient, I think the physician needs to bring it up to the patient as an early part of the discussion.

*Physician #6*

The most commonly cited strategies, however, were those that increased the salience of a clinical trial. Strategies included having a study champion (*n* = 2), discussing patients on a regular basis (*n* = 5), and setting up automated reminders (*n* = 6). Physicians agreed that revisiting patient information and being consistently reminded about available trials were effective strategies for ensuring patients were considered for participation.

R: So often we’ll, so we so there are two ways, one is, one is we meet every week to discuss patients and their care to make sure everything is moving on track and whether there are options for certain patients. And, and I think presenting the background at our rounds or something is the useful thing.

*Physician #8*

R: Right. It’s probably like it’s gonna we have to have our clinical trials people involved so that they’re aware and they can flag people for us at any points. But generally speaking I think this is going to be a patient population that we’re already treating and are failing our therapies. So, reminders are very helpful any kind that every time we have someone come by and give us a reminder on our clinical trial it tends to improve accrual. So, you know, email reminders or, you know, someone just a small update on accrual, the newsletters all those things that people use for trials I think are helpful in terms of reminding us.

*Physician #13*

# Social Influence

***N =* 14**

Physicians screening behaviours were primarily influenced by their colleagues (*n* =11). How and when physicians seek input from their colleagues depends on how a site is structured. For sites where hematologists are part of a tumour board, decisions regarding trials and screening are made as a group. Decisions were made first when a board considers participating in a trial (*n* = 3). The board evaluates whether a trial is methodologically sound and whether recruitment is feasible at their site. Once the decision is made to participate in a trial, participants indicated that all physicians would be motivated to screen and recruit.

R: Well I mean we can, every centre is obviously different. But in the lymphoma group we always discuss as a group each potential trial opportunity before even deciding to pursue that opportunity. And so, it’s discussed as a group to see whether or not it fits what our needs are and whether or not there’s a competing trial. Or if it’s something that we don’t think is either scientifically interesting or ethical. Beyond that if there is interest that’s sort of decided as a consensus by the group then we will pursue a trial. And so, I think many of those questions that you have about this particular issue are probably mitigated because we already have buy-in at that point.

*Physician #9*

Once a trial has been adopted by a site, those who worked with tumour boards or groups described meeting and discussing the eligibility of potential participants (*n* = 4). One physician indicated that their group also sought out input from nurses and social workers when screening patients for clinical trials.

R: Yeah I would envision it going that way, but you know, we also have a weekly Tumour Board where all new patients are presented or all patients requiring initiation of new therapy are presented. So that’s definitely an opportunity to capture potential candidate patients for enrolment.

*Physician #15*

Regardless of how a site is structured, physicians indicated that their colleagues influenced whether they screened a specific patient (*n* = 5). In some cases, the lead members from tumour boards or hematology groups were highly influential. Other physicians described deferring to experts in a specific disease subtype when considering whether a patient should be screened. a couple suggested they were influenced by their colleagues in so far as they sought out information from them but that their decision to screen was their own.

But you may run that case by your colleagues and say if a colleague thought, you know, perhaps because of their experience with the same trial that oh their patient did poorly on this because this was not right etc., then it might help me counsel the patient a little bit better but I don’t think it would influence my decision to enrol the patient in a trial or not. *Physician #5*

In addition to colleagues, six physicians indicated that patients and caregivers would influence their decision to screen in so far as their views were taken into consideration:

R: So, I think the, the pressure is likely to be, you know, a patient comes in with, you know, a desperate situation and they may, you know, they may benefit from CAR T-cells or somebody thinks they may benefit from CAR T-cells. We may get pressured to, to screen that patient for the study, right? We may, we may get called and told you know what, you know, maybe this is somebody you might want to consider? I’m not sure if that counts as, as pressure?

*Physician #1*

One physician indicated they took many considerations into account including nurses, fellows, patients and their families. Another physician indicated they were not influenced by anyone.

# Skills

***N* = 12**

Doctors indicated that screening patients for clinical trials was primarily learned on the job (*n* = 7) and did not usually require training that was in addition to residency medical training and specialization (*n =* 4). Only one doctor, who was also a clinical trialist, indicated having completed additional, formal training. They did, however, suggest that clear protocol guidelines were essential for proper screening and that screening improved with opportunities to screen multiple patients (*n =* 6). Two physicians indicated that protocol guidelines would be important given that CAR-T cell therapy is relatively new.

R: Probably I would I mean I would imagine it’s, it’s a little bit unique. We’re not just talking about a typical pharmaceutical agent or, you know, so I think there would probably need to be a bit of additional training of the involved team.

*Physician #15*

Two doctors discussed the importance of attending to patient cues and catering information delivery to the individual needs of a patient.

# Emotion

***N* = 13**

More than half of the hematologists in this study expressed enthusiasm for the upcoming CAR-T clinical trial. Despite expressing concerns, many (*n* = 8) were excited about the possibility of a new treatment options for their patients:

R: Well, you know, I think the, you know, I, I’m as susceptible to enthusiasm as the next guy and I really, really, really want to try CAR T-cells and I think my patients really, really want me to try CAR T-cells when they need them. So, I think that enthusiasm would, you know, drive a lot of patients in, you know, to be would drive me to screen a lot of patients.

*Physician #1*

When asked about the role of emotions during the screening process, physicians indicated that emotions were neither a barrier nor an enabler. One specifically indicated that emotions would not impact the screening process. Others (*n* = 9) suggested that having a new treatment option to offer patients who had just received bad news was advantageous. The stated that delivering bad news to patients was a common part of their job and that it would not dissuade them from discussing the CAR-T trial in the same conversation.

So, the, the really hard and tragic situation is when you’re breaking bad news and part of the bad news is that you have nothing left to offer them to try and get rid of their leukemia. And that’s devastating particularly if were they to be living in the States or in Europe or in a different kind of geographical location they may have potential of cure with access to CAR T-cells. So if I, so I, you know, breaking bad news I think it’s always important if you have the option of considering another treatment that’s the time to, to, you know, once it’s a realistic possibility that’s the time where I prefer to introduce it.

*Physician #11*

Two physicians did indicate that they might feel reluctant to discuss a clinical trial with patients but did not elaborate:

I: So, for example if let me give you a situation. If there’s a tense situation and the patients receive some bad news how would that influence your screening?

R: Hmm yeah that will affect the screening process as well, but whenever they come into the clinical trial they hope that it work.

*Physician #10*

I: So, I actually wanted to ask you are there any situations that come up where you might feel reluctant in speaking with a patient about a potential study?

R: Hmm the answer has to be yes. I’m trying to think of examples because again you’re probably getting the vibe I love clinical trials [laugh]

*Physician #12*

# Optimism

***N* = 12**

Of the participants that responded, most (*n* = 10) were optimistic about the trial. Two participants remained cautiously neutral suggesting that the point of the trial was to determine whether outcomes were more favourable than not and that ultimately, it was up to patients to decide whether the trial offered more good outcomes than bad.

I: But then would you then say recruiting or enrolling a patient into this trial will have more positive outcomes than negative?

R: Absolutely, absolutely like it give, it gives patients something to hope for that they don’t have right now at least not readily. –

*Physician #2*

R: Well I think CAR T-cells themselves are hugely promising.

*Physician #12*

R: That is the reason for this study, so you wouldn’t prejudge that. On the other hand, you wouldn’t enter patients into an intentionally risky treatment if you didn’t already have some indication that there would be some benefit. So, what that benefit and risk ratio is, is part of the study, of course, so we would not have a problem with that.

*Physician #7*

# Beliefs about Capabilities

***N* = 11**

Of those who were coded into this domain, six indicated that they would be confident in screening patients for a CAR-T clinical trial.

I: And lastly, this is again going to sound a little bit interesting, but how confident are you that you could screen patients for a trial of CAR T-cell therapy?
R: I am 100% confident [laugh]

*Physician #15*

Another three physicians indicated that they would feel more confident if they had more information regarding the safety and efficacy of CAR-T cell therapy as well as more information regarding the trial study design.

R: I think so long as I believe that a trial has been designed well and is being conducted will and its safety of patients is paramount then I’m comfortable raising it. And if people say no that’s fine, but I, I have no qualms about offering discussion about possible clinical trials.

*Physician #12*

Only one physician indicated they did not feel confident in screening for a CAR-T trial citing doubts about the study design and feasibility.

R: Yeah so, you know, so late study, a slow study, and a narrowly too narrowly focused, too early a study, would make it difficult to look the patient in the eye and say well this is gonna be just as good as the expensive stuff from Novartis. I don’t know, yeah, I don’t know.

*Physician #7*

Finally, one participant suggested they may experience challenges in identifying specific types of patients because they would be referred to their centre rather than directly identified by unit staff.

R: So, patients themselves are referred from other centres because for lymphoma patients basically we don’t treat them upfront. So, these patients should be referred from other centres that treat lymphoma if they are refractory for this. So, this, so identifying patients we would be able only to identify these patients that are referred to us. While for acute leukemia it, it’s because we treat, we are the centre that treats this disease, so we’d be able to identify patients that are potential candidates.

*Physician #4*

# Nature of the Behaviour

***N* = 6**

Given that most physicians had not screened for a CAR-T clinical trial, many could not speak from experience. However, one hematologist suggested that screening for CAR-T therapy would likely be similar to the process of screening patients for allogenic transplants. Two indicated that they had identified patients for CAR-T trials happening in the United States and another two indicated that generally, screening procedures go well given the clearly articulated procedural guidelines.

Finally, one participant indicated they did not see screening for clinical trials as a routine behaviour since the diseases in question are rare while another physician suggested that once a doctor is familiar with a patient, they can determine eligibility. Screening then becomes a formality rather than a necessity. This suggests that screening may be automated and routine for some doctors but not others.

I: to what extent would screening become a part of your daily routine?

R: I don’t know, this is not it wouldn’t. This is not a daily issue these are rare diseases or uncommon diseases let’s say.

*Physician #7*

R: If you know them you have treated them the screening might be just, just the process but you might be able to identify the patient that this is a real potential subject and the screening is formality for this patient.

*Physician #4*

# Reinforcement

***N* = 2**

One physician shared a negative experience of screening a false positive and the other indicated that financial incentives were not needed to encourage screening practices.
